# Bear bile use at the intersection of maternal health in Cambodia

**DOI:** 10.1186/s13002-020-00380-6

**Published:** 2020-05-24

**Authors:** Elizabeth Oneita Davis, Mhairi Gibson, Thona Lim, Jenny Anne Glikman

**Affiliations:** 1 San Diego Zoo Institute for Conservation Research, 15600 San Pasqual Valley Rd, Escondido, CA 92026 USA; 2 Department of Anthropology and Archaeaology, University of Bristol, 43 Woodland Rd, Bristol, BS8 1UU UK; 3 Free the Bears, PO Box 723, Phnom Penh, Cambodia

**Keywords:** Bear bile, Cambodia, Illegal wildlife trade, Kinship, Maternal health, Uterine ailments

## Abstract

**Background:**

The consumption of bear gallbladders and bear bile in Southeast Asia is a persistent threat to bear populations. As part of a larger effort to understand the characteristics of bear part consumption in Cambodia, we uncovered a consumer base of women seeking treatment for post-partum and uterine ailments.

**Methods:**

To better understand this aspect of consumption, we interviewed 122 women in seven different provinces in Cambodia, probing into the motivations and influences for using bear bile, as well as what types of ailments Cambodian women use it for.

**Results:**

We found that it is generally used by young or expecting mothers, and for such issues as post-partum “fatigue” (*toas* in Khmer), which could encompass post-partum depression. A desire to be supported by kin networks seems to facilitate the continued use of bear gallbladder and bile for these purposes.

**Conclusions:**

We suggest that efforts to reduce consumption should focus on encouraging older kin to change their means of support to Western/biomedical and by extension non-wildlife alternatives.

## Introduction

The alternative treatment strategies that some mothers embrace can diverge in a plurality of ways from the dominant medicinal system. For example, mothers may embrace religion as a means of coping with their pregnancy, and there appears to be a positive health basis for doing so [1]. Similarly, traditional medicine can be turned to for treatments that may not be addressed by the Western medicinal system, such as the use of steam for “general recovery” post-partum, among the Khmer [2]. Turning to these alternatives may provide additional health benefits and may give the mother greater agency over her pregnancy, a process which arguably threatens “women’s autonomy” [3]. Although the Khmer generally strongly trust Western medical doctors (Davis et al. unpublished data), there may be (and almost certainly are) desires for control over pregnancy that supersede the treatments prescribed by Western medicine physicians.

The dominant ethnic group of Cambodia is the Khmer, who belong to the Austroasian language group and consequently are believed to be one of the “initial” groups to settle Southeast Asia [4, 5]. A long history of interaction and flow across Southeast Asia means that the Khmer have been influenced at various points and in varying degrees of intensity by India, China, and the other states of Southeast Asia, from the kingdoms in Vietnam to the many societies and kingdoms of Indonesia [4, 6, 7]. The adoption of Theravada Buddhism in the Angkor period cemented the agrarian Khmer society into a relatively stable and unchanging structure centered around the divine kings and the Buddhist pagodas, arguably until the French colonization years, despite the decline of Angkor in the late middle ages (approximately the fifteenth century AD) [4]. To this day, modern Khmer village life is centered around the pagodas of the village (e.g., [8]), and the majority of Khmer continue to be small scale rice farmers [9]. However, in southern Cambodia in particular, this traditional structure is now fighting against perhaps the most striking change yet—the pervasive and ubiquitous impact of Chinese development. This development has negatively impacted southern Cambodia in a variety of ways, one of which is the destruction of village stability in affected areas through such challenges as environmental impacts and loss of income [10]. In some cases, the village itself is partially destroyed (E. Davis, pers. obs.). The modern situation for Cambodians, particularly those in the south, can therefore be complex, uncertain, and constantly evolving [11]. This lack of certainty will in turn undoubtedly influence the decisions and practices of young Khmer mothers throughout the country.

In Cambodia, traditional medicine (TM) has a long and well-developed history, although it is significantly waning in dominance in the broader substrate of society [12]. TM persists in Cambodia, due to individual medical pluralism, which is the adoption and use of multiple medical systems. In Cambodia, this can constitute Western medicine, the use of TM, and the consultation of spirit mediums (the *boramey*) [13]. Khmer TM is similar to traditional Chinese medicine and traditional Vietnamese medicine in its conception of “humoral balance,” i.e., a “hot and cold” balance within the body [2]. Treatments are therefore designed to correct this balance, with hot ailments such as fever prescribed a cold medicine, such as bear bile [14]. Khmer TM (hereafter TKM) is generally noted to stem from Ayurvedic medicine, brought over from India between the ninth and fifteenth centuries, with the incorporation of ancient and endemic Khmer techniques ([15, 16], Ki Buhang, National Center for Traditional Medicine, pers. comm). The ancient Khmer kings of the Angkor era integrated TKM in the kingdom by building hospitals [17], and TKM continued apace until the entry of the French in the mid to late 1800s. The French brought Western medicine into the country, although as Trankell and Ovesen [18] note, such measures can have “very little positive effects on the health of the native populations.” However, the French diverged from other colonizing powers by making the Khmer (as well as the other French “subjects” in Indochina) the focus of their medical efforts [19]. This had mixed success; the colonial government implemented the system but did not educate the greater populace about how Western medicine differed from TKM. Consequently, “Cambodians were the most hesitant to go” to the first “fully equipped” hospital in Phnom Penh in the early twentieth century [19]. Despite such disconnects, throughout the course of their colonization the French government trained a substantial number of Cambodians in Western medicine and actively built Western medical infrastructure such as hospitals and pharmacies [19]. In the present day, it is therefore no stretch to state that Western medicine now has a 100+ year history in the country and is thoroughly integrated. This has held true despite the turmoil of the Khmer Rouge, where the Western medicine system was largely destroyed and substituted for a medicinal system that was neither fully TKM nor fully Western [20]. As with all other aspects of post-Khmer Rouge Cambodia, the pre-Khmer Rouge Western medicinal system had to be rebuilt again; yet crucially, the Cambodians already understood and valued Western medicine, which was a marked difference from the Cambodia of the late 1800s [19, 20].

In Cambodia, traditional medicine treatments are both plant and animal-based, with animals such as rhinos (*Rhinocerotidae* spp.), slow loris (*Nycticebus* spp.), and bears (*Ursus thibetanus* [Asiatic black bear] and *Helarctos malayanus* [sun bear]) all consecrated in the traditional Khmer pharmacopeia (T. Lim, pers. obs.). The use of some of these wildlife products for medicinal purposes has been noted in present-day Cambodia (bears [21] and loris [22]). However, a deep understanding of Cambodian medical pluralism, and why and when certain TM products may be used as opposed to Western medicine, is lacking. This is important in this context for understanding the choices Khmer women may make around pregnancy, the rationale for these choices, and how this may impact maternal health in Cambodia. Understanding use of wildlife is also important from a biodiversity conservation standpoint, as wildlife populations decline across Southeast Asia, largely due to illegal and unsustainable trade and consumption [23, 24]. Additionally, understanding the specific medicinal reasons underlying demand for wildlife will inform thoughtful and targeted demand reduction campaigns [25], which in turn can compassionately incorporate the medicinal concerns and desires of the target group. Working towards the utilization of a non-illegal, non-wildlife alternative is therefore both a conservation and health priority.

Although bear bile/gallbladder is widely used across East and Southeast Asia for a variety of ailments [21, 23, 26], there are currently no published, peer-reviewed articles that document its use in women’s health. Here, our focus is on bear bile’s use by Khmer women in Cambodia for uterine, pregnancy, and post-partum ailments. Asiatic black bear bile has a medicinal basis, with a long history of use in China for hot ailments such as fever, general pain, inflammation, and epilepsy [14]. Currently, bear bile continues to be used for medicinal purposes in China, with one recent study estimating the prevalence of use at nearly 30% of the Beijing sample [27]. In Vietnam, which shares medical similarities with China, bear bile appears to be used more often and is generally prescribed for much the same ailments, with the most common being bruising, general pain, and fever [28]; however, bear bile as a post-partum treatment was rarely be cited by Vietnamese respondents. In Cambodia, bear bile is also used for bruising, fever, and general pain and is estimated to be used by about 15% of individuals, over the course of their lifetime [21]. Additionally, the use of bear bile in countries like Cambodia is interesting from a medicinal standpoint due to the greater numbers of sun bears as opposed to Asiatic black bears. Currently, there is little medical research into whether sun bear bile is as effective as Asiatic black bear bile, but it is suspected that it does not have the same medicinal efficacy (Davis et al., forthcoming).

## Methods

**Fig. 1:**
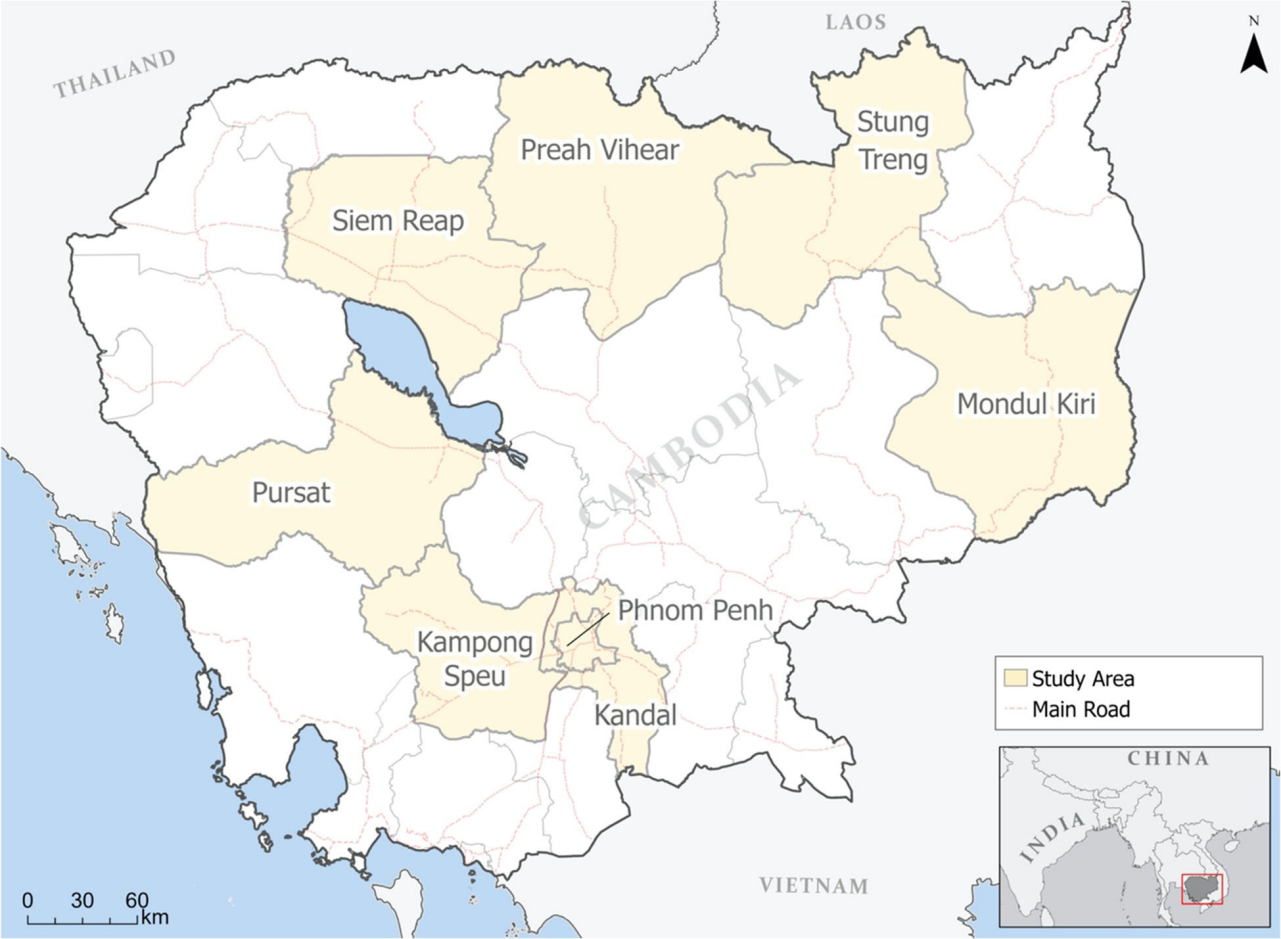
A map of Cambodia, with the provinces where interviews were conducted shaded in light yellow (map created by J. Stacy-Dawes)

Using semi-structured interviews with Cambodian women across the country (Fig. 1), we present a picture of bear bile use for pregnancy and post-partum ailments and analyze the rationales for its use, the potential drivers of its use, and further research avenues. By employing an ethnographic, female-centered approach, we have been able to both document this hitherto undetected use and gain important insight into its mechanics. Through understanding this previously unexplored use of a wildlife product, we believe this study provides an important contribution to greater understanding of how Khmer women “strategize their reproductive choices” in a challenging environment [29], and by extension how this strategization can be managed and addressed in a manner that benefits women while preserving bear populations within Cambodia.

Through semi-random and convenience sampling, we interviewed 122 women throughout Cambodia, in seven provinces that all differed in their ethnic makeup and level of development (Table 1). We performed interviews over two different time periods. In 2016, we performed interviews in Phnom Penh City (*n* = 42), Kandal Province (*n* = 7), and Kampong Speu Province (*n* = 7). In 2018–2019, we performed interviews in Mondulkiri Province (*n* = 13), Preah Vihear Province (*n* = 14), Pursat Province (*n* = 5), Siem Reap town (*n* = 9), and Stung Treng province (*n* = 14). Specific demographic information is not reported here due to the variable sampling strategies. In the sampling strategy of the 2018–2019 data collection, older women and women who were pregnant were specifically targeted. In addition, no demographic information was collected other than age and ethnicity. Age is not reported here due to the skew in the later data period of older women being one of the target groups. However, in Table 1 below, we provide the number of specific ethnicities represented (e.g., Khmer-Lao) in each province. To ensure that we targeted the Khmer, only the women who identified as Khmer or Khmer-Chinese/Lao/etc. are represented in this document. We targeted the Khmer because they are the predominant group in Cambodia. As a result, we have situated our interpretation of our findings within Khmer history, society, and practices. In the initial data collection period, we collected data on respondents’ religions and found that the majority of respondents were Theravada Buddhist (*n* = 40/42), an unsurprising result in light of Theravada Buddhism’s dominance within Cambodia [30]. Although we did not collect data specifically on religion during the second data collection phase, we expect that religious preferences were similar.

**Table 1:** Provinces in which interviews were conducted, with associated explanatory characteristics of urbanity and ethnic makeup, and specific ethnic characteristics of the women interviewed

Province	Characteristics	Ethnicities represented
Phnom Penh*	The urban center of Cambodia. Well-developed and predominantly Khmer.	Khmer (*n* = 23), Khmer-Chinese (*n* = 19)
Kandal*	The province just east of Phnom Penh City. People who work in Phnom Penh often commute in from the villages of Kandal. There are also many garment factories and, recently, significant Chinese development (E. Davis, pers. obs.).	Khmer (*n* = 4), Khmer-Chinese (*n* = 3)
Stung Treng	The least populated province situated near the Laos border. Many Khmer here identify as partially Lao.	Khmer (*n* = 9), Khmer-Lao (*n* = 5)
Kampong Speu*	The province in between the Cardamom Mountains and Phnom Penh. It is semi-rural, comprised mostly of farmland and clothing factories. The people are predominantly Khmer.	Khmer (*n* = 4), Khmer-Chinese (*n* = 3)
Mondulkiri	Well-forested and amenities are under-developed. Many indigenous groups live in this area, such as the Bunong.	Khmer (*n* = 12)
Pursat	Adjacent to the Cardamom Mountains, which is one of the few remaining biodiversity enclaves in Cambodia. It is well-connected and predominantly Khmer.	Khmer (*n* = 4), Khmer-Chinese (*n* = 1)
Preah Vihear	A reputed wildlife trade hotspot situated near a protected area. It is predominantly Khmer.	Khmer (*n* = 12), Khmer-Chinese (*n* = 2)
Siem Reap	Well-developed town and predominantly Khmer.	Khmer (*n* = 4), Khmer-Chinese (*n* = 4), Khmer-French (*n* = 1)

*Sites where interviews were conducted in Fall 2016. Interviews were conducted at all other sites throughout 2018 - 2019

We used multiple interview guides. Interview Guide A (Additional file 1) was used in Phnom Penh, Kampong Speu, and Kandal (denoted with a star next to their names), while Interview Guide B (Additional file 2) was used in all other sites. The variability in guides was due to two different stages of fieldwork, with the first stage conducted in Fall 2016, and the second throughout 2018–2019. For the second stage, the original instrument was refined and shortened, to target use of bear bile specifically, rather than all bear products.

The interviews in Phnom Penh and Kampong Speu were all led by the lead author, with the assistance of a translator. All other interviews were conducted by a trained team of Khmer women. The work was granted ethical approval by Miami University Ohio IRB for Human Subject Research (Protocol Number FWA00023676) and the University of Bristol Ethics Board.

## Results and discussion

### Familial relations in pregnancy

Although males can certainly be active participants in the pregnancy and post-partum process, in Khmer society it is generally women who devote their time and care to expecting and post-partum mothers [2]. These kinship networks are highly important for reducing possible anxiety associated with childbirth, and in particular anxieties that may arise during the post-partum period [31]. This is true across societies, including among the Khmer [2, 31]. However, such support naturally brings with it an additional measure of reliance. Older Khmer women urge their young female kin to perform a variety of traditional actions that are intended to ensure the health of mother and child. These include *chipon*, where a post-partum mother steams her face to improve her complexion upon giving birth, as well as *ang pleung*, where a mother “roasts” her body over hot coals placed below a slatted bed [2]. There is therefore pressure upon mothers to perform these actions, both to appease their female kin and to alleviate possible anxieties around the pregnancy process. However, it is worth noting that Khmer women are willing to take Western medicine in lieu of *ang pleung*, if it is available [2]. Nonetheless, the choice to take Western medicine over *ang pleung* is almost certainly still dependent on the blessings of the female kin.

### “People forced it on my sister”

One of EOD’s interviews into general bear part use in Phnom Penh was with a 19-year-old (known as I-57), who was able to answer in fluent English. It was in this interview that we first heard about the use of bear gallbladder for post-partum purposes.

My sister and aunty were given gallbladder after giving birth. People forced it on my sister.

But Western [medicine] is better, don't have to take gross, disgusting things after birth. Just rest for a week and [then you are] up and walking.

The young woman went on to say that it was her older female relatives, particularly her grandmother, who encouraged this use of bear gallbladder. Her brief snapshot of this process illuminates a struggle between Khmer generations, a lack of enthusiasm in the younger generation for the consumption of bear gallbladder, and a corresponding trust in Western medicine (hereafter biomedicine) over traditional treatments. This is likely especially so for those Khmer who have more money and opportunities, as I-57 clearly did, considering her fluency in English at a relatively young age. Those Khmer will have the means to pay for better healthcare than many others, and by extension they will have greater trust in the biomedicinal system to effectively care for them. This contrasts with the older females in the family, who will have experienced the turmoil of the Khmer Rouge and post-Khmer Rouge years, when there was no biomedical health system at all.

The war and post-war years resulted in more fully ingrained alternative treatment tactics among Khmer women and within these distinct kin networks. The Khmer Rouge obliterated both the TM and biomedical health systems that had existed, replacing the Western health system with ineffective facilities, supplies, and staff, while enacting strictures that severely limited the abilities of the traditional Khmer medicine practitioners. During the Khmer Rouge, death from neglect was common. Although Cambodians now generally trust biomedicine (Davis et al. forthcoming), which implies some measure of trust in the biomedical system now established in Cambodia, the legacy of self-reliance still resonates with Khmer mothers and their kin.

### Bear product wine for post-partum fatigue and pregnancy

In Kampong Speu, a provincial town approximately 2 h from Phnom Penh, there is a bustling market along the highway. It was here that an interview with one of the market women turned into a broad discussion with many other of the women selling their goods at the market. Initially passive observers, they became vocal when bear bile wine for treating post-partum “fatigue” was discussed. Post-partum fatigue, or *toas*, can encompass a plethora of ailments including headaches, abdominal cramps, and diarrhea [32], as well as potentially less easily defined issues including post-partum depression.

When discussing the use of bear bile wine to treat post-partum fatigue, all of the market women in the discussion advocated their support for this use, with proclamations of its efficacy. An interesting component of this widespread and unequivocal belief in efficacy among these women was that like many Khmer, they had little disposable income and little financial stability. They stated that the price of the bear bile wine they purchased (at that very market) was affordable. However, wealthy Phnom Penh elites suggested that those more affordable bear bile wine vials were likely all fakes, with actual bear bile/gallbladder being 100 times more expensive.

Although it has never been previously recorded as being used among Khmer women for this purpose, the use of bear bile wine is not without medical precedent in Khmer TM. The *ang pleung* process can be substituted by consumption of wine, as is or with animal and/or herbal products mixed within it [2]. The perception of efficacy of the product, however, is intriguing because the real likelihood that the market women were all consuming fakes correspondingly means that the wine may not have had biomedical efficacy. The widespread perception that it worked tells us that beyond the “placebo effect,” the support of the kin and in this case friend network is what resulted in the efficacy. The chain then was as follows: the kin network encouraged the use of bear bile wine by the mother, which the mother took, thereby physically affirming her place in the network and her acknowledgment of the advice and support of this group, which resulted in the strengthening of bonds between the actors, and stabilization if not increase in support and care from this network. A woman who diverged from the norm and refused to take bear bile would lose much of this valuable support. This holds true for the other bear products stated to be used for post-partum fatigue, such as bear bone wine. Bear bone in particular is unlikely to have little practical, biomedical health benefit for the woman. It is possible that for some other ailments bear bile/gallbladder is used for, such as “pushing the blood out” after giving birth, could be biomedically effective, but clinical research should be undertaken to assess the biomedical efficacy of this treatment. Generally, women tended to be vague about what exactly they were treating with bear products, beyond “pregnancy/post-partum,” and vague about what about the products gave them efficacy. This is not surprising, as it reflects a more holistic view of medicine and the body, which characterizes medicinal systems throughout East and Southeast Asia [13].

Intriguingly, the use of bear bile for post-partum care directly following the birth may conflict with established Khmer TM beliefs around a mother’s early post-partum state. Bear bile is a “cold” treatment intended for use in addressing “hot” ailments such as fever [14]. However, previous studies that investigated Khmer TM belief found that although the pregnancy period is a “hot” period where bear bile would theoretically be a sensible treatment, the post-partum period is a “cold” period, and thus only “hot” treatments should be prescribed [33]. Nonetheless, 13 out of the 22 women interviewed who stated that bear bile is a treatment for post-partum ailments (59%) indicated that the use of bear bile should occur in the “cold” early period following pregnancy, rather than later in the post-partum period (i.e., 6 months after giving birth). A 58-year-old woman in Mondulkiri said as follows:

post-partum illnesses: [bear bile] helps early mother to produce more and nutrition milk for feeding baby, having beautiful skin, and able to eat everything that they want without concerning the problem.

A 77-year-old woman in Pursat corroborated this statement as follows:

[bear bile/gallbladder is] good for women who just giving child birth because it can help her quickly heal the wound and recover well.

Respondents did not expand on this apparent disconnect between established Khmer TM beliefs and such use. As this use transcends across sites, it cannot be dismissed as a behavior localized to one community. In addition, bear bile is otherwise used in Cambodia in generally the manner expected, e.g., for fevers and bruising [13], so bear bile is not ordinarily used for cold ailments in the country. We speculate that this apparent disparity could be due to the use of wine, which on its own is considered “hot”; therefore, consuming any type of wine in the “cold” period after birth may be considered beneficial. However, more research is certainly needed into how bear bile/gallbladder wine can straddle both hot and cold ailments.

Despite the pressure Khmer women may receive from their kin to use bear bile/gallbladder, they can express some reservations around using it. In Stung Treng province, where there is generally a high level of bear bile/gallbladder consumption [13, 21], a pregnant young woman was given gallbladder by an older female relative, yet when interviewed she expressed opposition to the use of bear products generally. Her concerns were founded on the decline in bear populations, as well as the strictures of Buddhism which are opposed to killing animals. Yet, young pregnant women with such concerns who have bear bile/gallbladder pressed upon them by their female kin will ultimately not have a choice in what decision they make. The importance of the kin support network, and the possibility that such a product may indeed aid in their pregnancy and the post-partum period are strong advocates for consuming the product.

Beyond this dominant influencer of the support network, older Khmer women’s verbal arguments for using bear bile/gallbladder in particular are likely highly influential on those women who are “on the fence” about consuming wildlife, e.g., women who are not particularly enthused about wildlife consumption. Said one older Khmer woman as follows:

For pregnant women or early mother, it can help to them to feel better, for tonic, and can help to avoid any illnesses involve to post-partum (kind of illnesses that involve to early mother has eaten something wrong that make her sick) because bear eats different kind of foods so that’s why the women who use the bear gallbladder during her pregnant or post-partum can eat everything without worries it will make them sick.

This presentation of bear gallbladder as a panacea that can stave off illness and ensure health would be seductive to an expecting mother concerned about her pregnancy. Paired with the guarantee of the assistance of her female kin, there is absolutely no reason not to consume bear bile/gallbladder.

Another woman, meanwhile, stated as follows:

Mostly, people buy bears from Ratanakiri [a forested province in Cambodia]. I heard that we can take out the gallbladder from the bear without having to kill them. I also heard that gallbladder can grow again in bear’s bodies.

This shows that the woman who took the medicine believed that it would not be concurrently harming a bear. Had she known that doing so would cause the death of a bear, she may have expressed the same concerns as other women who expressed reservations around their use. However, she would likely have continued using it, for the reasons discussed above.

A final note around use of bear product wine for pregnancy/post-partum ailments is that bear products are not at all limited to this use. Older women across Cambodia had taken bear blood wine as a means of discharging menstrual blood, and other women had taken it to relieve cramping symptoms. In general, bear products appear to be consecrated as a treatment option in the sphere of Cambodian women’s uterine issues.

### Future research

One component worth exploring in future studies is that of the nebulous term of “post-partum fatigue” or *taos*. White [33] noted that although there were multiple forms of *taos*, it generally seemed to refer to “acute illnesses,” including dysentery and severe abdominal pain. The slightly euphemistic translation of “fatigue” may encompass the reality that young mothers are generally fatigued, particularly so for Khmer women who must also perform household duties and even work in the rice fields, in addition to caring for a newborn, which can then result in more severe illnesses. However, it is also worth noting again that this term can also encompass post-partum depression [19], which at present is largely understudied among Asian women [31]. This could again be where efficacy of bear bile/gallbladder wine is perceived by these mothers, as the support network facilitates and encourages use of bear product wine, while also providing practical care and aid during the challenging first few months, when the diverse symptoms of *taos* can first occur.

Another fascinating point for future exploration is the disconnect between the use of bear bile wine to treat “hot” ailments such as fever and bruising in Cambodia, as well as to treat ailments such as fatigue that can arise during the “cold” early post-partum period. At present, we speculate that this disconnect is due to an overriding belief in wine’s “hot” properties; however, research that explores the validity of this hypothesis would be useful. More generally, the health effects of these bear products on women should also be explored, considering the noted health risks of some Khmer TM treatments [33, 34]. This could be one of many challenges that the Cambodian government and/or non-governmental organizations could address to positively impact women’s health, particularly in rural areas where medical services are lacking, and the role of the kin support group is even more necessary.

In conjunction with the above, organizations could evaluate whether individuals would be willing to switch to Khmer TM herbal treatments for the post-partum period, and if such a switch would negatively impact flora in Cambodia. Under Cambodian law, foraging for herbal medicine can occur in protected areas [35]; however, there have been few if any rigorous assessments of foraging impact. In addition, the evaluation aspect of research into a switch to herbal medicine is critical, as previous efforts in Vietnam to switch bear bile consumers to herbal alternatives appear to have been largely unsuccessful due to these consumers perceived relative lack of herbal medicine efficacy [28].

Finally, beyond bear bile, there is also a lack of data around other illegal wildlife products which may also be used for uterine issues, with the same possible implications of pressure on wildlife populations. This is a critical research avenue for gaining a more complete understanding of the scale and scope of illegal wildlife trade and use in Cambodia, and for identifying wildlife species in need of comparable mitigation efforts. It is entirely possible that this particular conservation issue is greater than we currently see.

## Conclusion

In modern Cambodia, the legacies of the past and the realities of the present affect Khmer women’s choices. We show here that this environment has encouraged some women to choose the use of bear bile/gallbladder as uterine and/or pregnancy-related treatment. This is highly contingent on the specific kin network, yet if that kin network is sufficiently vocal and encouraging about use of bear products, this advocacy can supersede any other concerns (such as conservation or religion) that Khmer women may have.

Ultimately, young Cambodian women in particular seem to be on the cusp of a generational flip. At present, they continue to listen to their kin support network and subconsciously or consciously believe that this network is important for their health and the health of their child. However, projected estimates of greater affluence for Cambodians as the country’s economy continues to grow [36], with accompanying continued improvement in the “Western” biomedical system, will give future Cambodian mothers a greater support network outside of their female kin. This will encourage the women who already perceive some disadvantages of consumption to feel greater empowerment in rejecting the advice of their kin to use bear products.

Finally, as bears are declining throughout Southeast Asia and certainly in Cambodia, it is imperative that demand for their products is reduced. Although previous estimates are of bear product consumers comprising approximately 15% of the population [21], there is potential for this use to increase, considering at least 50% of the population in Cambodia is “at risk” to use bear products. We show here that conservation and governmental agencies should not attempt to reduce demand for bear bile through vilifying the actions of women who take bear products, particularly as we recognize that the uterine-related health issues that women face may be beyond the care that biomedical doctors can currently give, both in and out of Cambodia, due to a lack of research into these issues, and a corresponding lack of approved medicines and treatments [37, 38]. However, the lack of clinical data on bear bile’s effectiveness in treating uterine ailments does not suggest that it is a good option for women. Despite possible issues with sustainable harvest and/or perceived efficacy, traditional Khmer herbal medicines and alternative treatments such as steaming and *ang pleung* could be embraced, considering the lack of biomedical treatments available for some of the ailments discussed here. However, to more generally begin reducing use of bear bile, we suggest that older women should be encouraged to act as influencers in a positive way, through providing support for their kin that facilitates *ang pleung* or biomedical health care, rather than bear bile or other wildlife consumption. Through these means, young women can continue to feel supported and confident in their health and the health of the baby, while also mitigating pressure on threatened bear populations in Cambodia.

## Supplementary information

**Additional file 1.** Interview Guide A

**Additional file 2.** Interview Guide B

## Data Availability

All data is available on request. The interview guides used can be found in the Supplementary Materials.
