# Supplementary material for: Bear bile use at the intersection of maternal health in Cambodia
Source: J Ethnobiol Ethnomed. 2020 May 24;16:28. doi: 10.1186/s13002-020-00380-6 (PMC7245845; doi:10.1186/s13002-020-00380-6)
Supplement: Supplementary file 1 — Additional file 1. Interview Guide A [file 13002_2020_380_MOESM1_ESM.docx]

1. What does it mean to you to be Khmer?
2. Do you identify with a religion? If so, do you believe that religion has influenced your views towards wildlife? Why or why not? How do hunters and fishermen figure in this?
3. How would you feel, if you encountered a bear?
4. Can you tell me any folk tales that you know of about bears, specific to Khmer culture?
5. How do you think bears are perceived in the culture you identify with?
6. Do you believe that your feelings about bears are different from the feelings your parents have/had?
7. Would you consider someone who used bear parts to be of high-status?
8. Why would you use bear parts? Why would you not use bear parts?
9. What do you perceive to be the cost of using bear parts? What do you perceive to be a benefit?
10. What medicine would you use if you got sick? (Western, traditional, or a combination?)
