# Supplementary material for: Bear bile use at the intersection of maternal health in Cambodia
Source: J Ethnobiol Ethnomed. 2020 May 24;16:28. doi: 10.1186/s13002-020-00380-6 (PMC7245845; doi:10.1186/s13002-020-00380-6)
Supplement: Supplementary file 2 — Additional file 2. Interview Guide B [file 13002_2020_380_MOESM2_ESM.docx]

1. ***Age:***
2. ***Gender:***
3. ***Ethnic group:***
4. ***Occupation:***
5. ***Where do you live now?*** :
6. ***Where have you lived most of your life?​***
7. ***What culture do you most Identify with?***

Health Care

# 8.As far you know, what types of healthcare are available in this commune?

# 9.If you were sick, what type of healthcare would you use?

# 10.Why do you use that type of healthcare?

# 11.[If you recommend multiple types of healthcare] In what contexts are different healthcare systems used? (e.g., disease/ accessibility/cost/tradition) ?

# 12.If someone in your family was sick, what type of healthcare would you recommend?

# 13.[If you recommend multiple types of healthcare] In what contexts are different healthcare systems used? (e.g., disease/ accessibility/cost/tradition) ?

# 14.Who do you think is the typical person who uses ONLY scientific/western medicine?

# 15.Do you think the proportion of people using scientific/western medicine has changed over the last 10 years?

# 16.[If YES] How and why do you think that is?

# 17.Who do you think is the typical person who uses ONLY TKM?

# 18.Do you think the proportion of people using TKM has changed over the last 10 years?

# 19.[If YES] How and why do you think that is?

# 20.Who do you think is the typical person who uses ONLY TCM?

# 21.Do you think the proportion of people using TCM has changed over the last 10 years?

# 22.[If YES] How and why do you think that is?

Wildlife use

# 23.Have you heard of bear parts being used in TKM?

# 24.If so, which ones and for what medical conditions?

# 25.Have you heard of bear parts being used in TCM?

# 26.If so, which ones and for what medical conditions?

# 27.How do you think people who use bear parts are perceived in your culture?

Interviewer Comments:
